# The legacy effect of synthetic N fertiliser

**DOI:** 10.1111/ejss.13238

**Published:** 2022-05-17

**Authors:** Wytse J. Vonk, Renske Hijbeek, Margaret J. Glendining, David S. Powlson, Anne Bhogal, Ines Merbach, João Vasco Silva, Hanna J. Poffenbarger, Jagman Dhillon, Klaus Sieling, Hein F. M. ten Berge

**Affiliations:** ^1^ Plant Production Systems Wageningen University and Research Wageningen The Netherlands; ^2^ CAS Department Rothamsted Research Harpenden UK; ^3^ SAS Department Rothamsted Research Harpenden UK; ^4^ ADAS, Gleadthorpe Research Centre, Meden Vale Mansfield UK; ^5^ Experimental Station Bad Lauchstädt, Department of Community Ecology Helmholtz Centre for Environmental Research Leipzig Germany; ^6^ Sustainable Intensification Program International Maize and Wheat Improvement Centre (CIMMYT) Harare Zimbabwe; ^7^ Department of Plant and Soil Sciences University of Kentucky Lexington Kentucky USA; ^8^ Department of Plant and Soil Sciences Mississippi State University Starkville Mississippi USA; ^9^ Agronomy and Crop Science Institute of Crop Science and Plant Breeding, Christian‐Albrechts‐University Kiel Germany; ^10^ Agrosystems Research, Wageningen Plant Research Wageningen University and Research Wageningen The Netherlands

**Keywords:** ^15^N, cereal production, fertiliser requirement, long‐term experiment, nitrogen recovery, nitrogen use efficiency, soil N retention, soil N supply, synthetic fertiliser N

## Abstract

Cumulative crop recovery of synthetic fertiliser nitrogen (N) over several cropping seasons (legacy effect) generally receives limited attention. The increment in crop N uptake after the first‐season uptake from fertiliser can be expressed as a fraction (∆RE) of the annual N application rate. This study aims to quantify ∆RE using data from nine long‐term experiments (LTEs). As such, ∆RE is the difference between first season (RE^1st^) and long‐term (RE^LT^) recovery of synthetic fertiliser N. In this study, RE^1st^ was assessed either by the ^15^N isotope method or by a zero‐N subplot freshly superimposed on a long‐term fertilised LTE treatment plot. RE^LT^ was calculated by comparing N uptake in the total aboveground crop biomass between a long‐term fertilised and long‐term control (zero‐N) treatment. Using a mixed linear effect model, the effects of climate, crop type, experiment duration, average N rate, and soil clay content on ∆RE were evaluated. Because the experimental setup required for the calculation of ∆RE is relatively rare, only nine suitable LTEs were found. Across these nine LTEs in Europe and North America, the mean ∆RE was 24.4% (±12.0%, 95% CI) of annual N application, with higher values for winter wheat than for maize. This result shows that fertiliser‐N retained in the soil and stubble may contribute substantially to crop N uptake in subsequent years. Our results suggest that an initial recovery of 43.8% (±11%, 95% CI) of N application may increase to around 66.0% (±15%, 95% CI) on average over time. Furthermore, we found that ∆RE was not clearly related to long‐term changes in topsoil total N stock. Our findings show that the—often used—first‐year recovery of synthetic fertiliser N application does not express the full effect of fertiliser application on crop nutrition. The fertiliser contribution to soil N supply should be accounted for when exploring future scenarios on N cycling, including crop N requirements and N balance schemes.

**Highlights:**

- Nine long‐term cereal experiments in Europe and USA were analysed for long‐term crop N recovery of synthetic N fertiliser.
- On average, and with application rates between 34 and 269 kg N/ha, crop N recovery increased from 43.8% in the first season to 66.0% in the long term.
- Delta recovery was larger for winter wheat than maize.
- Observed increases in crop N uptake were not explained by proportionate increases in topsoil total N stock.

## 1 INTRODUCTION

Long‐term experiments (LTEs), such as the Broadbalk Wheat Experiment at Rothamsted Research (UK), show that external nitrogen (N) inputs can increase crop yields by two or three times (Rasmussen et al., 1998). While ample N supply has led to increased food security in recent decades, it can cause environmental damage such as eutrophication of surface waters, loss of biodiversity and global warming. On the other hand, soil nutrient depletion, low yields and severe food scarcity are apparent in places with insufficient access to N inputs. Sustainable N management implies avoidance of excess application as well as avoidance of soil fertility depletion. This involves proper accounting for crop N requirements to meet given target yields, both in the short and long‐term. This is especially relevant in regions where drastic changes in fertiliser‐N input are advocated or expected.

Research on inorganic (also called synthetic or mineral) N fertilisers has largely focused on N uptake in the year of application, and recommendation systems commonly account only for first‐season effects. Relatively few studies have aimed at quantifying the long‐term effect of synthetic fertiliser N on soil N and crop N uptake, although the need has been recognised (e.g., by Thomsen et al., 2003). For organic manures, in contrast, long‐term increments of total soil N, soil organic matter (SOM) and crop N uptake are well documented (Lund & Doss, 1980; Schröder et al., 2005).

Yet, inorganic fertiliser N inputs may also change the size or composition of the soil N pool in the long‐term—directly or via crop residues—and this could potentially sustain an increased annual soil N supply and associated crop N uptake and yield (i.e., the legacy effect of synthetic fertiliser N application). While multiple‐year effects on soil N supply remain scarcely documented for synthetic N fertilisers, several estimations have been made in the UK based on trials with ^15^N‐labelling. Sylvester‐Bradley et al. (1987) calculated that 10% of synthetic fertiliser applied was re‐mineralised in the second year, 3% of the remainder in the third year, and 1% in each of the following years. Similar values were found by others who later followed the fate of fertiliser‐derived ^15^N over multiple years (e.g., Glendining et al., 2001; Macdonald et al., 2002; Smith & Chalk, 2018).

LTE's can be used to quantify long‐term apparent fertiliser N recovery, by comparing annual crop N uptake in plots that did or did not receive fertiliser N for many years. Long‐term recovery thereby accounts for both the continuous depletion—in the absence of fertiliser inputs—of initial soil N stock, and the possible build‐up of soil N under a regime of fertiliser input. Long‐term N response curves, therefore, show steeper yield responses to N (at low and moderate N rates) than curves from the 1‐year trials typically used to inform fertiliser recommendation systems (van Grinsven et al., 2022).

Trends in long‐term N recovery, as seen in LTEs, may provide an upper estimate of the effect that sustained inorganic N inputs may have on soil N supply and crop yield. In the Bad Lauchstädt trial (Germany), a long‐term increase in N recovery was observed between 1970 and 2016 (Figure S1). However, such trends do not necessarily reflect an increase in soil N supply caused by fertiliser input. They may also be caused by improvements in crop genotype or management or changes in climate. At LTE Ropsley (UK), Bhogal, Young, Sylvester‐Bradley, O'donnell, and Ralph (1997) found a positive trend in N recovery between 1978 and 1990. Interestingly, it seems that the positive trend in long‐term N recovery fraction overtime was steeper for higher N rates at Ropsley.

Other long‐term studies assessed the residual effect of historically applied N on current crop N uptake after changing N application rates (e.g., Maaz & Pan, 2017; Petersen et al., 2010; Thomsen et al., 2003). Petersen et al. (2010) studied several experiments in Scandinavia where a wide range of new N rates was superimposed on historical N rates. The effect of historical N rates was found to be small compared to the effect of the newly established N rates on crop N uptake.

In this study, we present a new analysis of the legacy effect of synthetic fertiliser N application, that borrows elements from the above‐cited studies. The objective of this study is to quantify the contribution of synthetic fertiliser N to soil N supply by evaluating the difference (*∆*RE) between short‐term recovery (RE^1st^) and long‐term recovery (RE^LT^) of fertiliser N in the aboveground crop biomass. We applied this method to a number of suitable cereal‐based LTEs. We hypothesized that the long‐term recovery of applied synthetic N is larger than the first‐season recovery and that this difference would be mediated by factors, including climate, crop type, experiment duration, average N application, soil clay content, calculation method for RE^1st^, and crop residue management.

## 2 MATERIALS AND METHODS

First, the existing literature was searched for LTEs with suitable experimental set‐ups (as described in Sections 2.1 and 2.2). Subsequently, RE^LT^, RE^1st^ and *∆*RE were calculated for a number of data sets within each LTE (Sections 2.3, 2.4 and 2.5). Finally, a meta‐analysis was conducted to find the mean *∆*RE and to explain observed variation using a number of co‐variables.

### 2.1 Data selection and criteria

Data were collected from journal articles that reported information about LTEs. The selection criteria for inclusion in this study were as follows: (1) at least one long‐term fertilised (NPK) and one long‐term unfertilised control (PK) plot should be present to quantify RE^LT^; (2) either a ^15^N or a new control (PK) subplot superimposed on the long‐term N‐fertilised plot is present to allow quantifying RE^1st^. Using the search terms ‘Long‐term’ and ‘Cereal’ and ‘^15^N’ and/or ‘subplot’ in Google Scholar and Web of Science, five useful experiments were selected. Another experiment was obtained from the CATCH‐C database which contains data from over 300 LTEs in Europe (Sandén et al., 2018). (None of the other LTEs in that extensive collection met our criteria). Via personal communication, three more useful experiments were found. In total, this resulted in nine useful LTEs, which contained data from 11 experimental sites. When data were not fully provided in an article, they were obtained either by personal communication or by analysing figures from the article using Webplotdigitizer (Rohatgi, 2020).

### 2.2 Characteristics of LTEs included in the meta‐analysis

**FIGURE 1:**
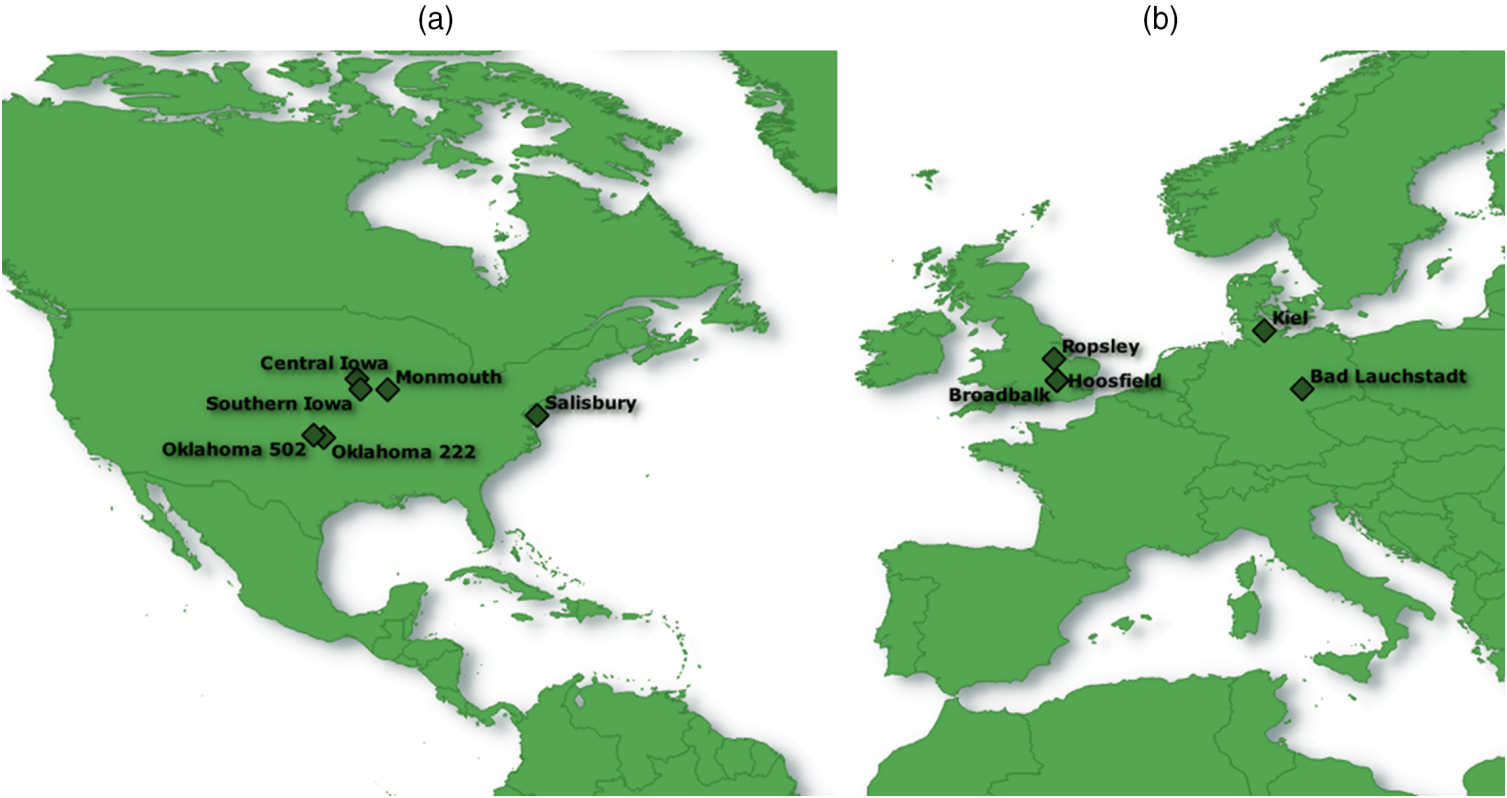
Locations of long‐term experiments in North America (a) and Europe (b) included in this study

The selected experiments suitable for the calculation of *∆*RE were located in Europe and North America (Figure 1). Crop residues (excluding roots and stubble) were removed from the field, except for the LTEs in Kiel and Iowa. The duration of the experiments varied between 5 and 141 years, with an average value of 38 years (Figure S2). No experiments were found with a duration between 21 and 80 years. Although a duration as short as 5 years may not be regarded as ‘long‐term’ by many, and while long‐term effects may indeed become more apparent over time, we still retained all data, considering the scarcity of LTEs that allowed assessment of both RE^1st^ and RE^LT^. An overview of *meta*‐data and slight deviations from the above methods to calculate *∆*RE is provided in Table 1. Such deviations include irreversible modifications in the experimental setup (instead of using temporary subplots) enabling the calculation of RE^1st^. A detailed description of all experiments is provided in Table S1.

**TABLE 1:** Descriptive information about the experiments that were used to calculate ∆RE

Name	Location	Crop type	Mineral fertiliser type	Duration experiment (years)	Sampling year(s)	Soil clay content (%)	RE^1st^ assessment method	Source
Broadbalk	Rothamsted Research, Harpenden, UK	WW	Calcium ammonium nitrate	138–141	1980–1983	28	^15^N	Powlson et al. (1986)
Ropsley	Ropsley, UK	WW	Ammonium nitrate	15 or 16	1992–1993	27	^15^N and subplot	Bhogal, Young, and Sylvester‐Bradley (1997)
Oklahoma‐222 and 502	Stillwater and Lahoma, OK, USA	WW	Ammonium nitrate	21 and 20, respectively	1989	20	^15^N. Because total N uptake was not measured in 1989, a value was calculated using crop yield as a reference from total N uptake in 1991.	Raun et al. (1999) and personal communication with Jagman Dhillon and Bill Raun
Monmouth	Monmouth, IL, USA	M	Urea	12	1994–1996	24	^15^N	Stevens et al. (2005)
Salisbury	Salisbury, MD, USA	M	Ammonium sulphate and ammonium nitrate	5	1977	15	^15^N	Meisinger et al. (1985)
Iowa‐central and southern	Ames and Chariton, IA, USA	M	Urea	16	2015	20 and 15 resp.	^15^N and subplot	Poffenbarger et al. (2018) and personal communication with Hanna Poffenbarger
Kiel	Kiel, Germany	WW & B	Calcium ammonium nitrate	7 or 9	1997–1999	15	^15^N	Sieling and Beims (2007) and personal communication with Klaus Sieling
Hoosfield	Rothamsted Research, Harpenden, UK	B	Calcium ammonium nitrate	119–121	1970–1972	20–25	Subplot method. However, instead of introducing a subplot a complete alteration of the experimental design was used to calculate RE^1st^ and RE^LT^ (Equation S1).	Rothamsted research (2015) and personal communication with Margaret Glendining
Bad Lauchstädt	Bad Lauchstädt, Germany	Rotation	Calcium nitrate	80	1979–1982	21	*∆*RE was directly calculated instead of first calculating RE^1st^ and RE^LT^ (Equation S2).	Körschens et al. (2002) and personal communication with Ines Merbach and João Vasco Silva.

*Note*: Crop type: WW, winter wheat; B, barley (winter barley in Kiel and spring barley in Hoosfield); M, maize.

### 2.3 Quantifying long‐term N recovery

**FIGURE 2:**
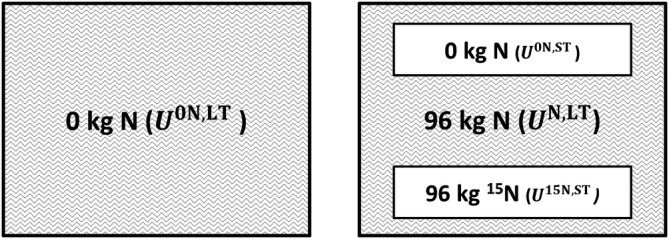
Examples of short‐term treatments within a long‐term trial which allow for the calculation of ∆RE. The two shaded large fields have continuously received either synthetic N fertiliser or no N fertiliser. Often such treatments are part of a larger setup with multiple N rate treatments. At the right, examples of a ^15^N treatment and of a new control subplot are shown superimposed on the original (long‐term fertilised) treatment. The ^15^N subplot receives the same N rate as the historic N rate, but now the fertiliser is ^15^N labelled. The numbers indicate the amount of applied synthetic N fertiliser (these are examples only). Between brackets is indicated what is measured on these plots, with notation corresponding to Equations ((1), (2), (3))

The long‐term N recovery fraction (RE^LT^) was defined as the fraction of annually applied fertiliser N recovered in the aboveground crop biomass. It includes recovery of fertiliser‐N applied in the current season, as well as recovery of previously applied fertiliser N, via uptake of fertiliser‐derived soil N built up in previous seasons. It is expressed as a fraction of the annual application rate. RE^LT^ was calculated based on LTEs where fixed levels of synthetic fertiliser N were maintained over many years (Figure 2; Equation 1). To calculate RE^LT^ from an LTE, at least one N application rate and a control plot must be present in the experimental set‐up. The control plot should have received zero N, with phosphorus (P) and potassium (K) application at the same rate as the fertilised plot. As this method takes a zero‐N treatment as a reference, it should be referred to as ‘long‐term *apparent* recovery’ (as opposed to labelled N recovery), but the term ‘apparent’ is omitted for brevity in the remainder of the text.

(1) $${\mathrm{RE}}^{\mathrm{LT}}=\frac{U^{N,\mathrm{LT}}-U^{0N,\mathrm{LT}}}{N\;\text{rate}},$$

with: *U* ^N,LT^, annual N uptake from the long‐term fertilised plot (kg N/ha). *U* ^0N,LT^, annual N uptake from the long‐term non‐fertilised (control) plot (kg N/ha). N rate, amount of N applied annually (kg N/ha) to the long‐term fertilised plot.

Note that both the N and 0 N treatments here refer to the long‐term treatments in the LTE that are still being continued, undisturbed by recent interventions made to assess first‐season recovery. Note also that first‐season recovery (Section 2.4) is part of RE^LT^.

### 2.4 Quantifying first‐season N recovery

Within the long‐term trial fields, two types of superimposed short‐term experiments were considered suitable to calculate RE^1st^: (1) introduction of an 0 N subplot (as illustrated in Figure 2, upper subplot); (2) synthetic fertiliser application with a ^15^N isotope (Figure 2, lower subplot). RE^LT^ and RE^1st^ were both calculated for the year in which such short‐term treatments were added. For reliable estimation of the difference between RE^1st^ and RE^LT^, it is imperative that these two variables refer to the same year of observation, thus eliminating errors due to annual variation. Hence, the long term treatments (control and fertilised) are to be continued unamended during the trial superimposed for the estimation of RE^1st^.

#### 2.4.1 Method 1: Introducing a subplot

Experiments with a newly introduced control subplot (only receiving PK application without synthetic N fertiliser), enable the calculation of RE^1st^ by subtracting measured N uptake in the control subplot from the N uptake in the long‐term main plot and dividing by the long‐term N application rate (Equation 2).

(2) $${\mathrm{RE}}^{1\mathrm{st}}\equiv \frac{U^{N,\mathrm{LT}}-U^{0N,\mathrm{ST}}}{N\;\text{rate}\;},$$

with: *U* ^N,LT^, N uptake from the long‐term fertilised plot (kg/ha). *U* ^0N,ST^, N uptake from short‐term non‐fertilised (control) subplot (kg/ha), that is, where the historic long‐term N rate was discontinued in the year of observation. N rate, amount of N applied annually (kg N/ha) to the long‐term fertilised plot (as in Equation 1).

#### 2.4.2 Method 2: Using ^15^N

Alternatively, most of the LTEs allowed the calculation of RE^1st^ from observations of first‐season ^15^N recovery from fertiliser labelled with the ^15^N isotope (Powlson et al., 1986). This approach assumes that the two isotopes (^14^N and ^15^N) undergo chemical and biological transformations in the same manner. The ^15^N taken up by the crop was divided by the amount of ^15^N applied:

(3) $${\mathrm{RE}}^{1\mathrm{st}}\equiv \frac{\;U^{15N,\mathrm{ST}}}{15N\;\text{rate}\;},$$

with: *U* ^15N^, ^15^N uptake (kg/ha/year). ^15^N application rate (kg N/ha/year).

### 2.5 Delta recovery (∆RE)

The main response variable in this analysis, delta recovery (*∆*RE), was introduced to express the legacy effect of long‐term synthetic N application on crop N uptake. We define ∆RE as the difference between first season N recovery (RE^1st^) and long‐term N recovery (RE^LT^) in above‐ground crop biomass, both measured in the same year.

**FIGURE 3:**
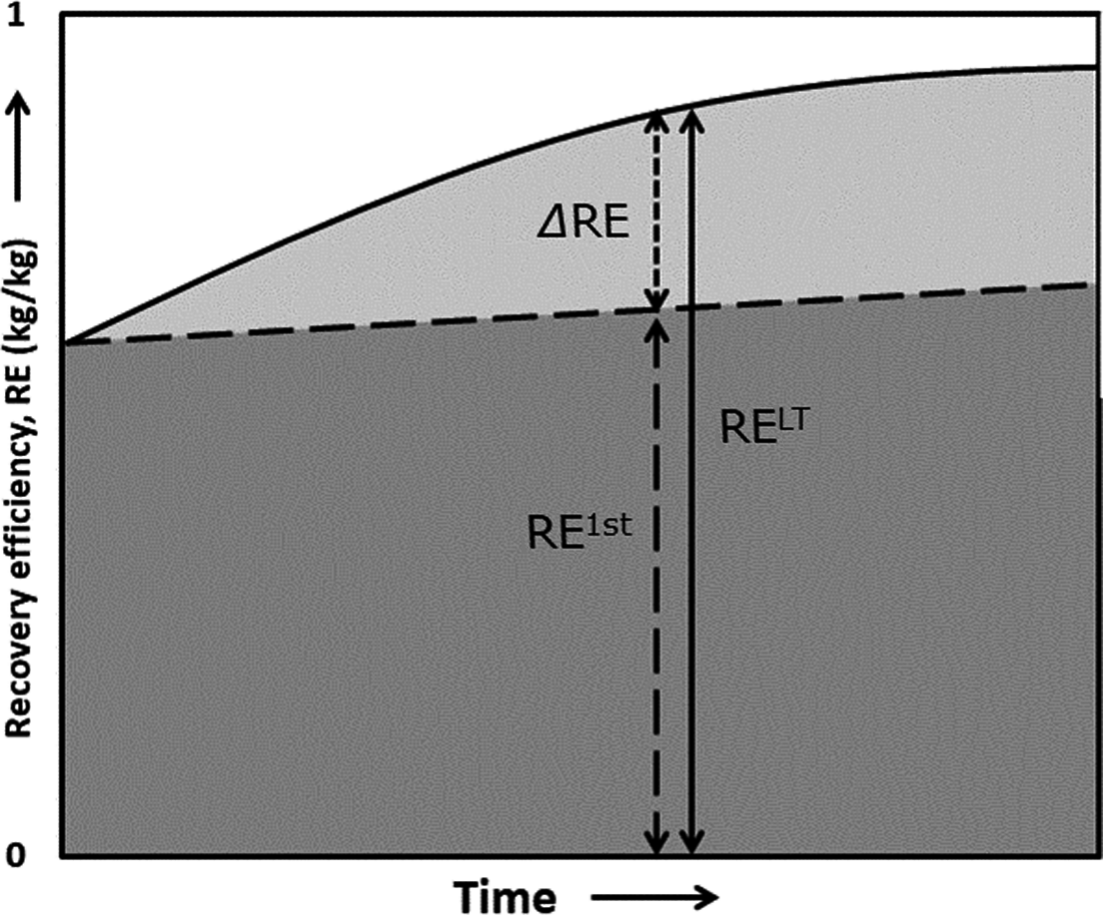
Hypothetical development of total N uptake and fertiliser N recovery, with continuous synthetic N application over time. The short‐term recovery fraction (dark grey) increases over time in this graph, possibly by long‐term improvements in, for example, cultivars or management. It could also decrease, for example, by changing biotic or abiotic stresses. The light grey area indicates the additional recovery (∆RE) of synthetic fertiliser N via increased soil N supply. The total N recovery in a certain year is called long‐term recovery (RE^LT^), indicated by the solid black arrow. Uptake from the native soil N stock (that existed prior to the start of LTE and dwindles over time) is not shown here

As explained, first season N recovery (RE^1st^) refers to the fraction of N taken up from fertiliser in the year of application (Figure 3, large dotted arrow). Long‐term N recovery (RE^LT^), in contrast, also includes uptake of N applied in earlier years (Figure 3, solid black arrow). The difference between RE^1st^ and RE^LT^ (∆RE, Equation 4) results from the uptake of fertiliser‐N that was retained in the soil and released beyond the year of application. Therefore, ∆RE could be thought of as ‘delayed N recovery’ and is expressed as the fraction of the annual N application rate (%).

(4) $$∆\mathrm{RE}\equiv {\mathrm{RE}}^{\mathrm{LT}}-{\mathrm{RE}}^{1\mathrm{st}}.$$

Total aboveground crop biomass was used to determine crop N uptake as a basis for calculating all recovery values (RE^1st^, RE^LT^, ∆RE). Cereal crops (wheat [*Triticum aestivum*], barley [*Hordeum vulgare*] and maize [*Zea mays*]) were the main focus of this study because these are the crops most commonly grown in long‐term studies.

### 2.6 Meta‐analysis

Relevant data from the nine LTEs were compiled in a database. Based on these, 66 sets of data were constructed, each of which allowed for calculating RE^1st^, RE^LT^ and ∆RE. This number (66) is larger than the number of LTEs (11), because data from multiple years, crop types and N application rates were available for some of the LTEs (Table S3).

Every observation included information about experimental location, year, N application rate, RE^1st^ calculation method and N uptake. Most studies included in this analysis did not provide a measure of variance for N uptake. Moreover, for some studies, every data point included only a single observation. Therefore, the number of underlying replicates was used as a weighting factor for the data points, including the number of years and the number of true field replicates. Experiment location and sampling year were included as random effects. For both RE^1st^, RE^LT^ and ∆RE, the normality of distribution was checked using density plots (Figure S3).

#### 2.6.1 Comparing RE^1st^ calculation methods

First, the extent to which the method used to calculate RE^1st^ (either ^15^N or Subplot method) affected ∆RE was tested. This comparison was performed both on the whole dataset, but also separately on a subset of the experiments where both methods were used. The latter gives the most straightforward comparison but can be applied to a few LTEs only. As this selection reduced the sample size and the other analyses were performed using all data points, the type of method was also added as an explanatory variable for ∆RE using a mixed‐effects model from the nlme package in R (Pinheiro et al., 2020) on the whole dataset.

#### 2.6.2 Calculation of mean ∆RE

To calculate a weighted average of ∆RE across all studies, a mixed effect model was used from the nlme package in R (Pinheiro et al., 2020). In cases where both methods to calculate RE^1st^ were available, a separate value for ∆RE was included for each of the two methods.

#### 2.6.3 Mixed‐effects model estimation and selection

Besides quantifying ∆RE, this study also aimed to quantify the influence of several co‐variables on ∆RE (Equation 5) such as crop type, experiment duration, average N application, soil type and climate. Co‐variables were standardised to the same unit to enable comparison between studies. The effect of co‐variables was tested in several combinations using mixed‐effects models. To find the combination of co‐variables that best fitted the data, a model selection was performed with the ‘dredge’ function from the Mumin package (Barton, 2019), based on the corrected Akaike information criterion (AICc). Models were considered to be different when ∆AICc >2. Fixed effects that were tested are provided in Equation 5. Co‐variable values were mostly obtained from the published articles. In addition, the climate zone for each LTE was characterised by the Global Yield Gap Atlas approach, defining three main features: growing degree days (accumulated temperature sums for mean daily temperature above a base temperature), aridity index (annual total precipitation divided by annual total potential evapotranspiration), and temperature seasonality (quantified as the standard deviation of monthly average temperatures) (Van Wart et al., 2013). The variable named ‘crop residue retention’ indicated whether crop residues (straw) were removed or kept on the field after harvest (the latter being the case in two LTEs). Information about other co‐variables is included in Table S3. However, additional variables were not included in the analysis because the data were not available for all experiments.

(5) ∆RE∼Growing degree days+Temperature seasonality+Aridity index+Crop type+Experiment duration+AverageNapplication+Soil clay content+Method+Crop residue retention+ε.

#### 2.6.4 Total soil N

In addition to the co‐variables shown in Equation (5), the relation between ∆RE and the long‐term change in total soil N stock was examined. As soil N data was only available for three experiments, this was done as a separate analysis. For those three experiments, the relative increase in total soil N (i.e., in fertilised plot versus unfertilised plot) was compared with the relative increase in soil N uptake (again in fertilised plot versus unfertilised plot; Equation 6).

(6) $$\text{relative soil}\;N\;\text{uptake increase}=\frac{∆\mathrm{RE}*N\;\text{rate}}{U^{0N}}*100\%.$$

## 3 RESULTS

### 3.1 Observed ΔRE across nine experiments

**FIGURE 4:**
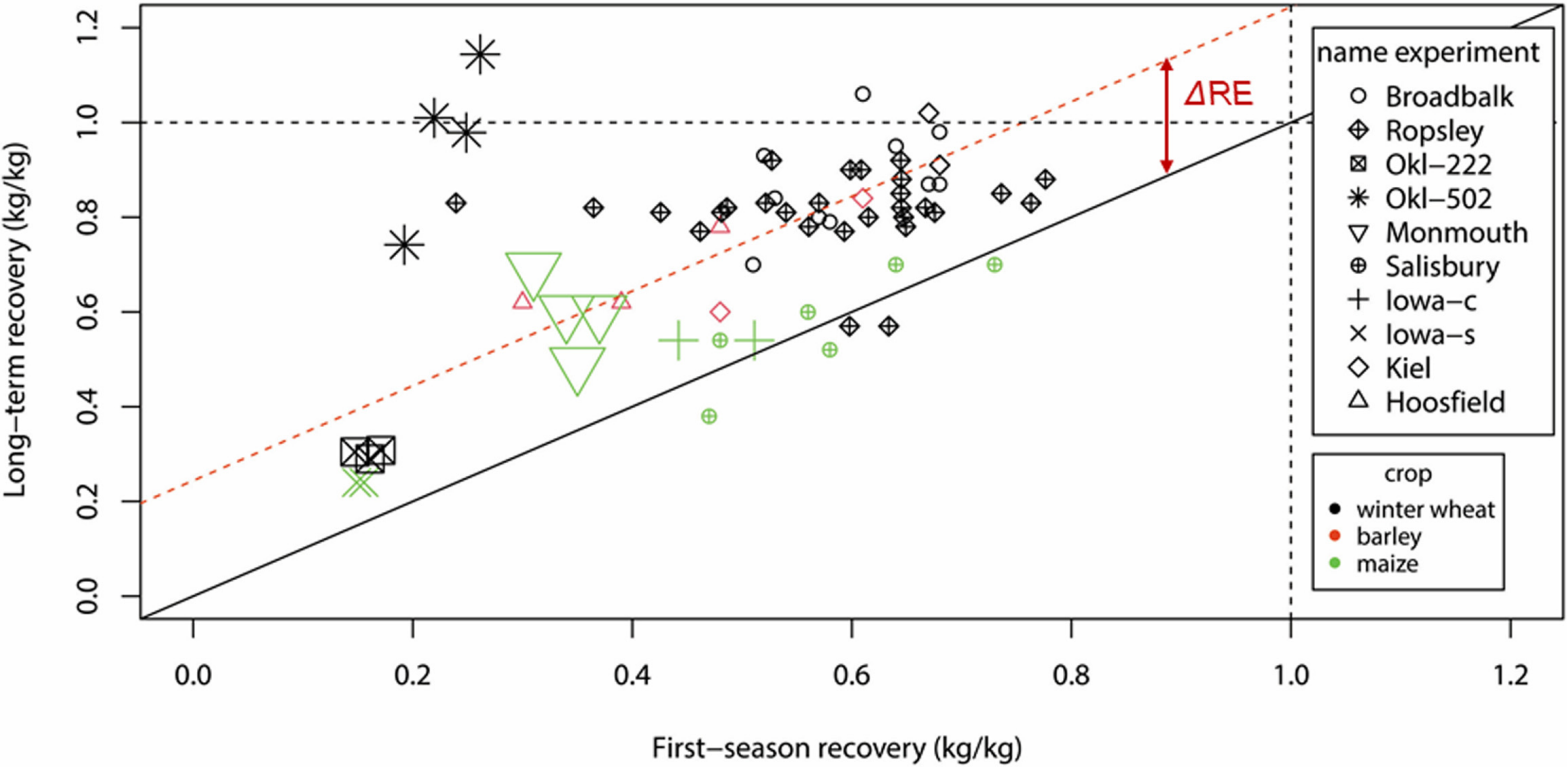
First season recovery and long‐term recovery for winter wheat, spring barley and maize (*N* = 66). The diagonal solid black line indicates RE^LT^ = RE^1st^; the difference between that black line and the red diagonal dashed line indicates the estimated average ∆RE. Point‐size indicates the weight based on sample size. Note that data from the LTE Bad Lauchstädt is excluded from this graph because separate values of long and first season recovery could not be calculated and ∆RE was assessed differently (see Table S1; see Equation S2)

In 61 out of 66 cases, RE^LT^ was larger than RE^1st^ (Figure 4). Mean RE^1st^ and RE^LT^ were 43.8% (±11%, 95% CI) and 66.0% (±15%, 95% CI) of annual N application rate, respectively. For four observations, RE^LT^ exceeded 100%, meaning that the increment in N uptake (over the control treatment) was larger than the amount of N applied. Such points were retained in the overall analysis. Across all data‐points, mean ∆RE was 24.4% (±12.0%, 95% CI) of the annual N application rate.

### 3.2 Influence of co‐variables on ΔRE

**FIGURE 5:**
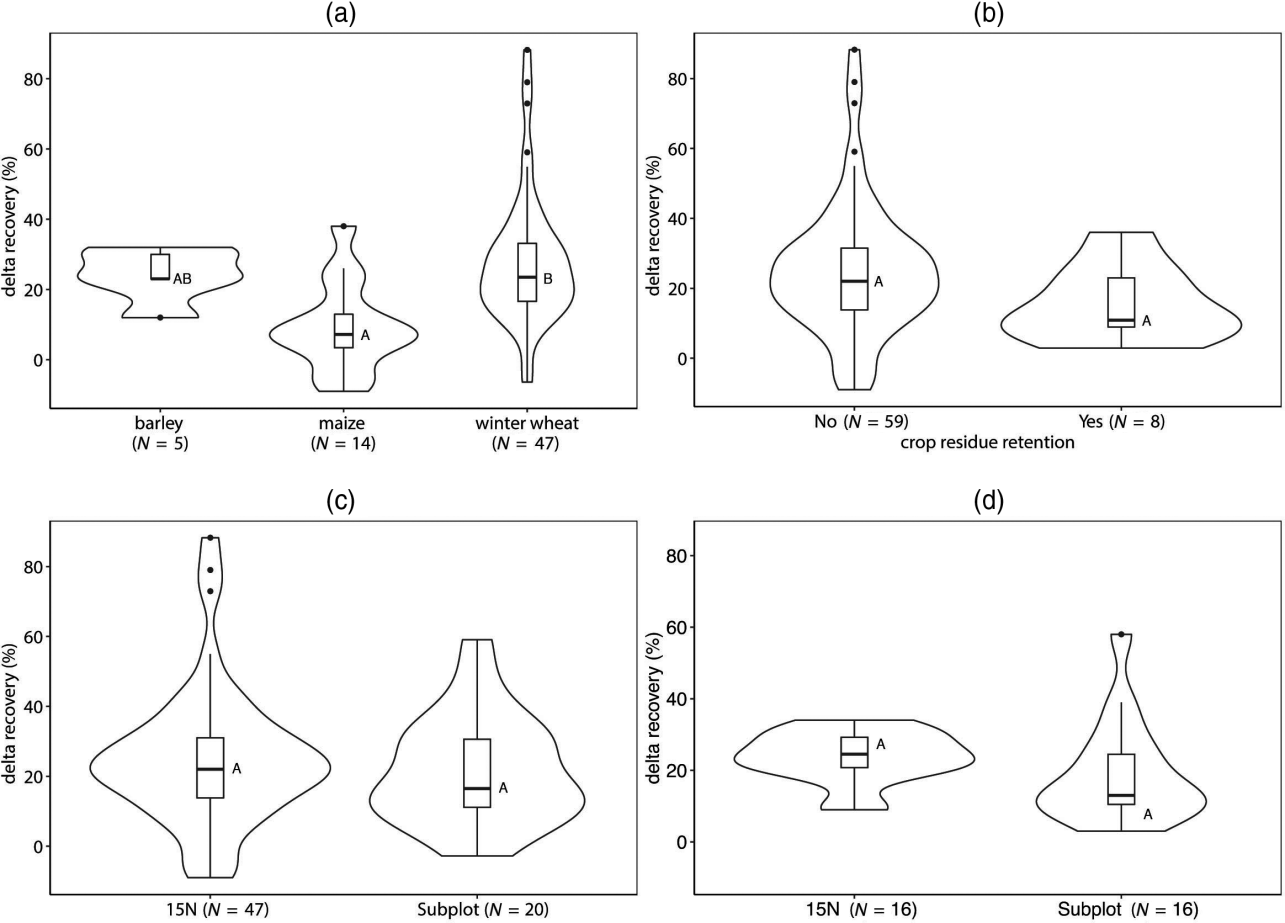
Violin plots of the distribution of ∆RE, separated for (a) crop types, (b) crop residue retention (no *N* = 59, yes *N* = 8), and (c,d) method; within each violin plot, a boxplot indicates the median, lower (1st) and upper (3rd) quantile of the data. Plot (c) illustrates the distribution of all data points (^15^N *N* = 47, subplot *N* = 20). Plot (d) contains only data from an experiment, which allowed for both ^15^N and the Subplot method (*N* = 16). If upper case letters near the median are identical then there is no significant difference

No severe collinearity was observed between the co‐variables included in the full model (i.e., including all co‐variables; further explained in Figure S4). The two models with the lowest AICc values include the variables crop type, method, and crop residue retention, with or without clay content (Table 2, Figure 5). Winter wheat showed a significantly higher ∆RE than maize (*p* = 0.002). ∆RE did not significantly differ from the other crop types. Soil clay percentage was included as a predictive variable for ∆RE in the second‐best model. However, the estimated slope of 1.07% of the annual N application rate per percent clay content was not significant (*p* = 0.45) when included as a sole variable. The effect of clay content on RE^LT^ seemed more evident but was not significant (*p* = 0.24, Figure S5). Crop residue retention was included in all selected models. However, when including crop residue retention as a sole variable, no significant difference in ∆RE (*p* = 0.38) was observed between retention and removal of crop residues. Crop residues were retained at two of the nine LTEs.

**TABLE 2:** Model results of a model without co‐variables, with all co‐variables and the four best models based on AICc model selection

Co‐variable	Only *∆*RE	Full model	Model 1	Model 2	Model 3	Model 4
Growing degree days	−	+	−	−	−	−
Temperature seasonality	−	+	−	−	−	−
Aridity index	−	+	−	−	−	−
Crop type	−	+	+	+	+	−
Experiment duration	−	+	−	−	−	−
Average N application	−	+	−	−	−	−
Soil clay content	−	+	+	−	+	+
Method	−	+	+	+	−	+
Residue retention	−	+	+	+	+	+
AICc value	558.1	584.2	545.2	545.5	548.4	548.5
*∆*RE estimate	24.4% (±12.0%, 95% CI)					

*Note*: ∆RE estimates are only given for the model without co‐variables because there are multiple estimates for the other models.

Including experiment duration and level of N application did not improve the AICc–value of any linear mixed effect model used to explain variation in ∆RE (Table 2). When assessing the effect of experiment duration and N application as a sole variable on ∆RE, no significant correlations were observed (Figure S2). As the correlation between duration and ∆RE may be more pronounced in earlier years (with RE^LT^ levelling off after some point in time), we also selected a subset of experiments with a duration between 5 and 21 years. In this period, no significant effect of duration could be found either (*p* = 0.399).

The type of method to assess RE^1st^ (^15^N or Subplot method) was included as an explanatory variable in the best model when using data from all experiments (Figure 5c). In the experiments that allowed for both methods to calculate RE^1st^ (Ropsley, Iowa‐central and Iowa‐southern, Figure 5d), ∆RE was not significantly different between both calculation methods (*p* = 0.18). However, when removing one outlier, the ^15^N method showed a significantly higher ∆RE than the Subplot method (*p* = 0.008). The difference between both methods amounted to a 7.4% recovery of annual N application, caused by lower RE^1st^ values from ^15^N treatments compared to the Subplot treatments.

In three out of nine experiments, total soil N data were available. Topsoil total soil N was, on average, 10% higher on fertilised plots compared to the control plots. N uptake from the soil in the fertilised plots, by contrast, increased by 86% on average, relative to unfertilised control (Figure S6). There was no significant correlation between the relative increments of N uptake from the soil on the one hand, and total soil N on the other (*p* = 0.41).

## 4 DISCUSSION AND CONCLUSION

### 4.1 Long‐term recovery is consistently higher than short‐term recovery

Our results show a consistently positive ∆RE, which indicates that N originating from earlier synthetic fertiliser applications contributes to crop N uptake in years after its application. Based on our data set, the mean legacy effect of synthetic N fertiliser application (∆RE) was 24.4% (±12.0%, 95% CI) of the annual N application rate. Figure 4 suggests that ∆RE may decrease with increasing values of RE^1st^. We therefore also assessed the legacy effect as a fraction of (1 − RE^1st^), rather than as a fraction of the N rate. However, expressing the legacy effect in this manner yielded no additional insights, as equally so, no correlations were found with explanatory variables (e.g., with experiment duration). Moreover, in practical cases, RE^1st^ is often unknown beforehand. Expressing ∆RE as a fraction of N application, therefore, has much more practical value. We do recognise, however, that in a true equilibrium state the sum of RE^1st^ and ∆RE cannot exceed 1 in situations where crop residues are removed from the field (as in most LTEs here).

Our observed ∆RE corresponds well with multiple ^15^N studies which followed the fate of a single synthetic ^15^N application over multiple years (e.g., Dourado‐Neto et al., 2010; Glendining et al., 2001; Macdonald et al., 2002; Smith & Chalk, 2018). Nitrogen retention was assessed in those studies by measuring the fraction of applied ^15^N that ends up in the soil pool or crop. Additionally, when following ^15^N in the soil for multiple years, the return to the soil of fertiliser‐derived N via crop residues was also measured in those studies. While that method allows following a ^15^N ‘spike’ (once applied) over several years, it does not allow quantification of the cumulative effect on crop N recovery of maintaining a fixed N rate over many years. Jenkinson et al. (2004) followed such a single ^15^N pulse for nearly 20 years in old grassland, where the grass was harvested every year. In the year of application, about 47% of applied ^15^N was recovered. Cumulated over the following 18 years of the experiment, another 17% of the initially applied ^15^N was recovered in aboveground biomass, which is quite similar to the mean value of an additional 24.4% recovery for cereals found in our study.

Glendining et al. (1996) also found evidence for a positive ∆RE, using the same method that is here called ‘Subplot method’, at Broadbalk where N application was withheld for one season. In the year that no N fertiliser was provided to the long‐term plots, N uptake was higher on those ‘withheld’ plots that had previously received synthetic fertiliser N, than on the long‐term control plot which had never received N fertiliser. The maximum additional N uptake compared to the long‐term N_0_ treatment was found to be 29 kg N/ha on a plot, which previously received 192 kg N/ha. The corresponding ∆RE value would be 15.1% of N applied. The interpretation of their results was somewhat difficult due to weed growth. Nonetheless, their reported value corresponds roughly to the value of ∆RE that was found in this study. (Our analysis included data from the same Broadbalk experiment, albeit from other years.)

In our study, weighting was used to give more importance to observations that were based on more replicates. However, weighting can also result in a bias towards those agro‐ecological conditions for which a higher number of replicates happened to be available. Nonetheless, the estimated mean ∆RE only increased marginally (0.5%‐point) when excluding weights in the mixed‐effects model. Furthermore, the model selection results did not change when weights were excluded.

### 4.2 Influence of crop type, soil clay content and RE^1st^ calculation method

Crop type, soil clay content, crop residue retention and RE^1st^ calculation method were the most important factors governing ∆RE, based on the model selection results. Winter wheat showed a significantly larger ∆RE than maize (Figure 5a), possibly because of its longer growing season, finer root system and ability to root deeper, which would enable it to use mineralised N from SOM more effectively (Thorup‐Kristensen et al., 2009). Two out of the three experiments that cultivated maize used urea as fertiliser type, while all other experiments used a variation of ammonium‐nitrate. There was no significant difference in ∆RE between the two types of fertiliser, neither when evaluated on the whole dataset nor when based on the maize subset only (data not shown). Soil type may also affect the ability to store and re‐mineralise fertiliser N. Soils with a higher clay content show a larger N retention capacity compared to sandy soils (Cheshire et al., 1999). However, both RE^LT^ and ∆RE were not significantly influenced by soil clay content in our study (Figure S5). Retaining crop residues on the field was suggested to play an important role, based on the model selection results. However, crop residue retention did not significantly influence ∆RE when included as a sole variable in the model. Crop residues were retained in only two experiments, admittedly too few to obtain a firm view of the effect of crop residues on ∆RE. Finally, the method used to estimate RE^1st^ (either ^15^N or Subplot method) was found to affect ∆RE. However, this was only significant within the subset of experiments where both ^15^N and the Subplot method were available to calculate RE^1st^, and only so when one outlier datapoint was removed.

^15^N experiments are known to underestimate RE^1st^ (Jenkinson et al., 1985; Quan et al., 2021), which would lead to higher estimates for ∆RE (the difference between RE^1st^ and RE^LT^ becoming larger). Underestimation of RE^1st^ could be caused by phenomena collectively referred to as ‘added nitrogen interactions’ (ANI). This includes pool substitution: labelled N replacing unlabelled N that would otherwise have been immobilised, leaving more unlabelled N available for plant uptake, and so causes overestimation of the contribution of soil‐N to crop N uptake (Jenkinson et al., 1985). Stepwise N rate experiments avoid these difficulties but are potentially afflicted with the ‘priming’ issue (i.e., synthetic N application increasing soil N mineralisation). Quan et al. (2021) mention that differences between ^15^N and Subplot methods tend to increase over time. However, with increasing experiment duration, no significant change in the difference between both methods was found in our data (*p* = 0.51, Figure S2). Differences between ^15^N and Subplot methods could also be caused by the ‘law of diminishing returns’, when the Subplot method uses different control N rates (Quan et al., 2021). The latter situation does not occur in our study: 0 N control plots were used in all experiments that relied on the Subplot method. Finally, more ^15^N experiments than ‘subplot’ experiments were found in the literature. This is most likely because ^15^N trials are less disturbing to the main setup of LTEs.

### 4.3 Limitations of this study

#### 4.3.1 Low variation in co‐variable values

Climate, which was included in the regression model by using growing degree days, temperature seasonality, and an aridity index, did not significantly contribute to the explanation of observed variation in ∆RE, even when accounting for the influence of other co‐variables. However, this could be due to the small variation in climate among the experimental locations. Most experimental sites were located around the same latitude, some with a more continental and others with a more maritime climate. For other climates, results may differ. However, no suitable long‐term experiments were found beyond temperate climates.

Somewhat surprisingly, the duration of the experiments did not affect ∆RE. A possible caveat is the lack of data points in this study with an experimental duration between around 20 and 80 years (Figure S2). When data points were clustered in two groups, the group with durations above 80 years (14 out of 67 observations) showed no higher ∆RE than the group with durations between 5 and 21 years (*p* = 0.24). A likely explanation is that contributions to crop uptake from remineralized N diminish over time, so the increase in ∆RE from later years will become progressively smaller.

Because the experimental setup required for the calculation of ∆RE is relatively rare, the total number of observations used in this study was limited (*N* = 66, based on nine LTEs). As discussed, the limited size of our data pool, and especially the limited number of experiments included, may have caused the lack in observed correlations between co‐variables and ∆RE, for example for the experimental duration. Moreover, the prevalence of co‐variables in our dataset did not have a balanced distribution. For example, in contrast to winter wheat, there are no observations for maize with an experimental duration of over 26 years (Table S2). Similarly, most experiments included only one crop type and either retained residues on the field or not. Such configuration can lead to confounding effects. To alleviate this problem we used a mixed‐effects model as is common in meta‐analyses. This corrects for observations from the same experimental site. Nonetheless, we only found a significant difference in ∆RE between winter wheat and maize in our data. This does not rule out possible other correlations which however could not be substantiated. More data points, with more variation in co‐variable factors, may be required to reveal other possible co‐variable effects.

#### 4.3.2 Total soil N

If a cumulative effect of synthetic fertiliser N on soil N supply exists beyond the years of application, one would expect to find evidence also in changing soil N stocks. Previous studies reported clear, but relatively small increments in total soil N with increasing N application (Glendining & Powlson, 1995; Macdonald et al., 1989; Petersen et al., 2010). Glendining and Powlson (1995) indicated that total soil N increased under higher synthetic N application, but mineralisable N increased proportionally more. This suggests that changes in the quality of the soil N pool are governing ∆RE, rather than an increased total N stock. A similar conclusion was drawn in another study by Glendining et al. (1996). Bhogal, Young, and Sylvester‐Bradley (1997) reported a ‘break point’ at around 150–160 kg N application, above which N recovery increased more proportional to total soil N compared to lower N rates. Those findings correspond well with outcomes from our study, where the relative increase in total soil N after long‐term synthetic N application is much smaller than the relative increase in N uptake from soil. This may imply that ∆RE reflects a change in composition rather than the size of the total soil N pool, as Glendining and Powlson (1995) already suggested. To our knowledge, there is still no conclusive explanation for this disproportionality.

### 4.4 Implications of this study

Despite the relatively small number of observations and the potential difficulties afflicting the assessment of ∆RE, it is clear that our results show that continuous, long‐term application of synthetic fertiliser leads to a higher N recovery of the applied synthetic fertiliser N, as compared to the first season recovery. Additionally, it seems that crop type is the most important factor governing this process (Table 2). More long‐term experiments, with larger variations in all co‐variables (e.g., assessing ∆RE in other situations, such as a tropical climate), can help to further develop an understanding of sustainable N cycling. The outcomes of this study suggest that a single year N recovery of 43.8% (±11%, 95% CI) in the total aboveground biomass can become, on average, 66.0% (±15%, 95% CI) over time due to N retention in the soil and its subsequent release. This is different from the simplified 50% (e.g., Lassaletta et al., 2016) that is commonly used currently, which does not consider fertiliser N retention by the soil.

Due to the legacy effect of synthetic fertiliser N application (expressed here as ΔRE), N yield response curves based on long‐term trials show steeper slopes than those based on short‐term trials. As also argued by Van Grinsven et al. (van Grinsven et al., 2022), this shift in slope should be taken into account in studies that seek to strike a balance between farm profit, food security and the environment. This is especially relevant in regions where N input rates are drastically changed. For example when grain output must steeply rise to feed a growing population such as in sub‐Saharan Africa, or when N inputs are reduced to mitigate water pollution or greenhouse gas emissions as in parts of Europe today.

## AUTHOR CONTRIBUTIONS

**Wytse J. Vonk:** Conceptualization (equal); Data curation (equal); formal analysis (equal); methodology (equal); writing – original draft (equal); writing – review and editing (equal). **Renske Hijbeek:** Conceptualization (equal); formal analysis (equal); methodology (equal); supervision (equal); writing – review and editing (equal). **Margaret J. Glendining:** Data curation (equal); writing – review and editing (equal). **David S. Powlson:** Data curation (equal); writing – review and editing (equal). **Anne Bhogal:** Data curation (equal); writing – review and editing (equal). **Ines Merbach:** Data curation (equal); writing – review and editing (equal). **João Vasco Silva:** Data curation (equal); writing – review and editing (equal). **Hanna J. Poffenbarger:** Data curation (equal); writing – review and editing (equal). **Jagman Dhillon:** Data curation (equal); writing – review and editing (equal). **Klaus Sieling:** Data curation (equal); writing – review and editing (equal). **Hein F. M. ten Berge:** Conceptualization (equal); methodology (equal); supervision (equal); writing – review and editing (equal).

## CONFLICT OF INTEREST

The authors declare no potential conflict of interest.

## Supporting information


**Data S1**. Supporting Information.

## Data Availability

Data is published as part of the supplementary information.

## References

[ejss13238-bib-0001] Barton, K. (2019). MuMIn: Multi‐model inference v.1.43.6. https://CRAN.Rproject.org/package=MuMIn.

[ejss13238-bib-0002] Bhogal, A. , Young, S. , & Sylvester‐Bradley, R. (1997). Fate of 15 N‐labelled fertilizer in a long‐term field trial at Ropsley, UK. The Journal of Agricultural Science, 129, 49–63.

[ejss13238-bib-0003] Bhogal, A. , Young, S. D. , Sylvester‐Bradley, R. , O'donnell, F. M. , & Ralph, R. L. (1997). Cumulative effects of nitrogen application to winter wheat at Ropsley, UK, from 1978 to 1990. The Journal of Agricultural Science, 129, 1–12.

[ejss13238-bib-0004] Cheshire, M. , Bedrock, C. , Williams, B. , Christensen, B. , Thomsen, I. , & Alpendre, P. (1999). Effect of climate and soil type on the immobilization of nitrogen by decomposing straw in northern and southern Europe. Europe Biology and Fertility of Soils, 28, 306–312.

[ejss13238-bib-0005] Dourado‐Neto, D. , Powlson, D. , Bakar, R. A. , Bacchi, O. O. S. , Basanta, M. V. , Cong, P. , Keerthisinghe, G. , Ismaili, M. , Rahman, S. M. , Reichardt, K. , Safwat, M. S. A. , Sangakkara, R. , Timm, L. C. , Wang, J. Y. , Zagal, E. , & van Kessel, C. (2010). Multiseason recoveries of organic and inorganic nitrogen‐15 in tropical cropping systems. Soil Science Society of America Journal, 74, 139–152.

[ejss13238-bib-0006] Glendining, M. , Poulton, P. , Powlson, D. , Macdonald, A. , & Jenkinson, D. (2001). Availability of the residual nitrogen from a single application of 15N‐labelled fertilizer to subsequent crops in a long‐term continuous barley experiment. Plant and Soil, 233, 231–239.

[ejss13238-bib-0007] Glendining, M. , & Powlson, D. (1995). The effects of long continued applications of inorganic nitrogen fertilizer on soil organic nitrogen–a review soil management experimental basis for sustainability and environmental quality (pp. 385–446). Lewis Publishers.

[ejss13238-bib-0008] Glendining, M. , Powlson, D. , Poulton, P. , Bradbury, N. , Palazzo, D. , & Ll, X. (1996). The effects of long‐term applications of inorganic nitrogen fertilizer on soil nitrogen in the Broadbalk wheat experiment. The Journal of Agricultural Science, 127, 347–363.

[ejss13238-bib-0009] Jenkinson, D. , Fox, R. , & Rayner, J. (1985). Interactions between fertilizer nitrogen and soil nitrogen—The so‐called ‘priming’ effect. Journal of Soil Science, 36, 425–444.

[ejss13238-bib-0010] Jenkinson, D. , Poulton, P. , Johnston, A. , & Powlson, D. (2004). Turnover of nitrogen‐15‐labeled fertilizer in old grassland. Soil Science Society of America Journal, 68, 865–875.

[ejss13238-bib-0011] Körschens, M. , Merbach, I. , & Schulz, E. (2002). 100 Jahre Statischer Düngungsversuch Bad Lauchstädt. Herausgeber UFZ‐Umweltforschungszentrum Leipzig‐Halle GmbH.

[ejss13238-bib-0012] Lassaletta, L. , Billen, G. , Garnier, J. , Bouwman, L. , Velazquez, E. , Mueller, N. D. , & Gerber, J. S. (2016). Nitrogen use in the global food system: Past trends and future trajectories of agronomic performance, pollution, trade, and dietary demand. Environmental Research Letters, 11, 095007.

[ejss13238-bib-0013] Lund, Z. F. , & Doss, B. D. (1980). Residual effects of dairy cattle manure on plant growth and soil properties 1. Agronomy Journal, 72, 123–130.

[ejss13238-bib-0014] Maaz, T. , & Pan, W. (2017). Residual fertilizer, crop sequence, and water availability impact rotational nitrogen balances. Agronomy Journal, 109, 2839–2862.

[ejss13238-bib-0015] Macdonald, A. , Poulton, P. , Stockdale, E. , Powlson, D. , & Jenkinson, D. (2002). The fate of residual 15 N‐labelled fertilizer in arable soils: Its availability to subsequent crops and retention in soil. Plant and Soil, 246, 123–137.

[ejss13238-bib-0016] Macdonald, A. J. , Powlson, D. S. , Poulton, P. R. , & Jenkinson, D. S. (1989). Unused fertiliser nitrogen in arable soils—Its contribution to nitrate leaching. Journal of the Science of Food and Agriculture, 46, 407–419.

[ejss13238-bib-0017] Meisinger, J. , Bandel, V. , Stanford, G. , & Legg, J. (1985). Nitrogen utilization of corn under minimal tillage and moldboard plow tillage. I. Four‐year results using labeled N fertilizer on an Atlantic coastal plain soil 1. Agronomy Journal, 77, 602–611.

[ejss13238-bib-0018] Petersen, J. , Thomsen, I. K. , Mattsson, L. , Hansen, E. M. , & Christensen, B. T. (2010). Grain yield and crop N offtake in response to residual fertilizer N in long‐term field experiments. Soil Use and Management, 26, 455–464.

[ejss13238-bib-0019] Pinheiro, J. , Bates, D. , DebRoy, S. , Sarkar, D ., & Team RC . (2020). Nlme: Linear and nonlinear mixed effects models. R package version 3.1‐150. https://CRAN.R-project.org/package=nlme.

[ejss13238-bib-0020] Poffenbarger, H. J. , Sawyer, J. E. , Barker, D. W. , Olk, D. C. , Six, J. , & Castellano, M. J. (2018). Legacy effects of long‐term nitrogen fertilizer application on the fate of nitrogen fertilizer inputs in continuous maize agriculture. Ecosystems & Environment, 265, 544–555.

[ejss13238-bib-0021] Powlson, D. , Pruden, G. , Johnston, A. , & Jenkinson, D. (1986). The nitrogen cycle in the Broadbalk wheat experiment: Recovery and losses of 15N‐labelled fertilizer applied in spring and inputs of nitrogen from the atmosphere. The Journal of Agricultural Science, 107, 591–609.

[ejss13238-bib-0022] Quan, Z. , Zhang, X. , Fang, Y. , & Davidson, E. A. (2021). Different quantification approaches for nitrogen use efficiency lead to divergent estimates with varying advantages. Nature Food, 2, 241–245. 37118466 10.1038/s43016-021-00263-3

[ejss13238-bib-0023] Rasmussen, P. E. , Goulding, K. W. , Brown, J. R. , Grace, P. R. , Janzen, H. H. , & Körschens, M. (1998). Long‐term agroecosystem experiments: Assessing agricultural sustainability and global change. Science, 282, 893–896. 9794751 10.1126/science.282.5390.893

[ejss13238-bib-0024] Raun, W. R. , Johnson, G. , & Westerman, R. (1999). Fertilizer nitrogen recovery in long‐term continuous winter wheat. Soil Science Society of America Journal, 63, 645–650.

[ejss13238-bib-0025] Rohatgi, A . (2020). WebPlotDigitizer v4.4. https://automeris.io/WebPlotDigitizer.

[ejss13238-bib-0026] Rothamsted Research . (2015). Hoosfield spring barley experiment plans and fertilizer treatments, 1968–2000. Rothamsted Research, Electronic Rothamsted Archive. 10.23637/rhb2-plans1968-2000-01

[ejss13238-bib-0027] Sandén, T. , Spiegel, H. , Stüger, H. P. , Schlatter, N. , Haslmayr, H. P. , Zavattaro, L. , Grignani, C. , Bechini, L. , D′Hose, T. , Molendijk, L. , Pecio, A. , Jarosz, Z. , Guzmán, G. , Vanderlinden, K. , Giráldez, J. V. , Mallast, J. , & ten Berge, H. (2018). European long‐term field experiments: Knowledge gained about alternative management practices. Soil Use and Management, 34, 167–176.

[ejss13238-bib-0028] Schröder, J. , Jansen, A. , & Hilhorst, G. (2005). Long‐term nitrogen supply from cattle slurry. Soil Use and Management, 21, 196–204.

[ejss13238-bib-0029] Sieling, K. , & Beims, S. (2007). Effects of 15N split‐application on soil and fertiliser N uptake of barley, oilseed rape and wheat in different cropping systems. Journal of Agronomy and Crop Science, 193, 10–20.

[ejss13238-bib-0030] Smith, C. J. , & Chalk, P. M. (2018). The residual value of fertiliser N in crop sequences: An appraisal of 60 years of research using 15N tracer. Field Crops Research, 217, 66–74.

[ejss13238-bib-0031] Stevens, W. , Hoeft, R. , & Mulvaney, R. L. (2005). Fate of nitrogen‐15 in a long‐term nitrogen rate study: II. Nitrogen uptake efficiency. Agronomy Journal, 97, 1046–1053.

[ejss13238-bib-0032] Sylvester‐Bradley, R. , Addiscott, T. , Vaidyanathan, L. , Murray, A. , & Whitmore, A. (1987). International Fertiliser society‐proceeding 263.

[ejss13238-bib-0033] Thomsen, I. K. , Djurhuus, J. , & Christensen, B. T. (2003). Long continued applications of N fertilizer to cereals on sandy loam: Grain and straw response to residual N. Soil Use and Management, 19, 57–64.

[ejss13238-bib-0034] Thorup‐Kristensen, K. , Cortasa, M. S. , & Loges, R. (2009). Winter wheat roots grow twice as deep as spring wheat roots, is this important for N uptake and N leaching losses? Plant and Soil, 322, 101–114.

[ejss13238-bib-0035] van Grinsven, H. J. , Ebanyat, P. , Glendining, M. , Gu, B. , Hijbeek, R. , Lam, S. K. , Lassaletta, L. , Mueller, N. D. , Pacheco, F. S. , Quemada, M. , & Bruulsema, T. W. (2022). Establishing long‐term nitrogen response of global cereals to assess sustainable fertilizer rates. Nature Food, 3, 122–132. 37117954 10.1038/s43016-021-00447-x PMC10661743

[ejss13238-bib-0036] Van Wart, J. , van Bussel, L. G. , Wolf, J. , Licker, R. , Grassini, P. , Nelson, A. , Boogaard, H. , Gerber, J. , Mueller, N. D. , Claessens, L. , & van Ittersum, M. K. (2013). Use of agro‐climatic zones to upscale simulated crop yield potential. Field Crops Research, 143, 44–55.

