# Supplementary material for: The legacy effect of synthetic N fertiliser
Source: Eur J Soil Sci. 2022 May 17;73(3):e13238. doi: 10.1111/ejss.13238 (PMC9415127; doi:10.1111/ejss.13238)
Supplement: Supplementary file 1 — Data S1. Supporting Information. [file EJSS-73-0-s001.docx]

**Supplementary information to: “****The legacy effect of synthetic N fertiliser”**

*European Journal of Soil Science*

Wytse J. Vonk^1^, Renske Hijbeek^1^, Margaret J. Glendining^2^, David S. Powlson^3^, Anne Bhogal^4^, Ines Merbach^5^, João Vasco Silva^1,6^, Hanna J. Poffenbarger^7^, Jagman Dhillon^8^, Klaus Sieling^9^, Hein F.M. ten Berge^10^

**Institutional affiliations:**^1^Plant Production Systems, Wageningen University and Research, the Netherlands.

^2^CAS Department, Rothamsted Research, Harpenden, Herts AL5 2JQ, UK.

^3^SAS Department, Rothamsted Research, Harpenden, Herts AL5 2JQ, UK.

^4^ADAS Consulting Ltd., Gleadthorpe Research Centre, Meden Vale, Mansfield, Notts NG20 9PF, UK.
^5^Experimental Station Bad Lauchstädt, Dep. Community Ecology, Helmholtz Centre for Environmental Research – UFZ, Germany.

^6^Sustainable Intensification Program, International Maize and Wheat Improvement Centre (CIMMYT), Harare, Zimbabwe

^7^Department of Plant and Soil Sciences, University of Kentucky, Lexington, KY 40546, USA

^8^Department of Plant and Soil Sciences, Mississippi State University, Starkville, MS 39762, USA

^9^Institute of Crop Science and Plant Breeding, Christian-Albrechts-University, Hermann-Rodewald-Str. 9, 24118 Kiel, Germany.

^10^Wageningen Plant Research, Wageningen University and Research, the Netherlands.

**Contact information:**Wytse Vonk, [wytse.vonk@wur.nl](mailto:wytse.vonk@wur.nl)

**Glossary and abbreviations**

| LTE | Long-term experiment |
| --- | --- |
| N | Nitrogen |
| ^15^N | Rare isotope of N which can be measured in e.g. plant biomass or soil and is used to study the faith of applied fertiliser N. Its background abundance in soil is approx. 0.37 atom%. |
| N-uptake | Uptake of nitrogen, either in aboveground crop biomass or grain only (kg N/ha). |
| N-recovery | Difference in N uptake compared to a unfertilised control plot, expressed as a fraction of annual fertiliser N application rate (kg/kg). |
| *RE*^1st^ | First season recovery, which implies the recovered fraction of current season applied fertiliser N (not from previous application), expressed as faction of annual application rate. |
| *RE*^LT^ | Long-term recovery, which implies the recovered fraction of applied fertiliser N, from current year applied fertiliser N and from previously applied fertiliser N, expressed as fraction of annual application rate. |
| *∆RE* | Delta recovery, the difference between first-season- and long-term recovery. |
| U^N, LT^ | Annual N uptake from long-term fertilised plot (kg N/ha). |
| U^0N, LT^ | Annual N uptake from long-term non-fertilised (control) plot (kg N/ha). |
| U^0N, ST^ | N uptake from non-fertilised (control) subplot (kg/ha), where the historic long-term N rate was discontinued (just one year prior to observation). |


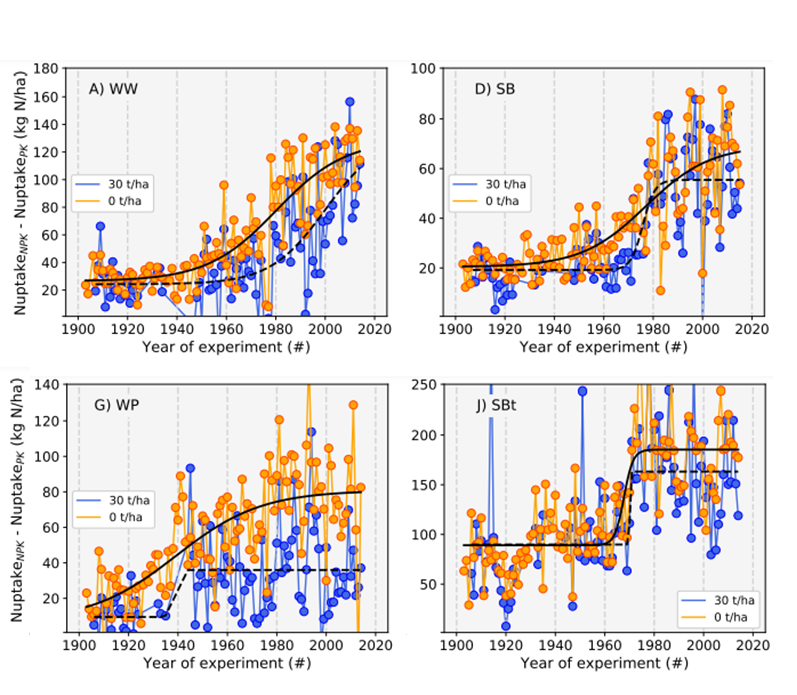


*SI-Figure 1: Long-term recovery development over time in Bad Lauchstädt for four crops (winter wheat, spring barley, ware potato and sugar beet), made by da Silva (unpublished). The blue and orange dots indicate the plots with and without added organic manure. From 1970 onwards, N application rates remained fairly stable but recovery still seems to increase. This is partly due to improvements in e.g. cultivars, management or climate, but additionally soil N retention could play a role.*


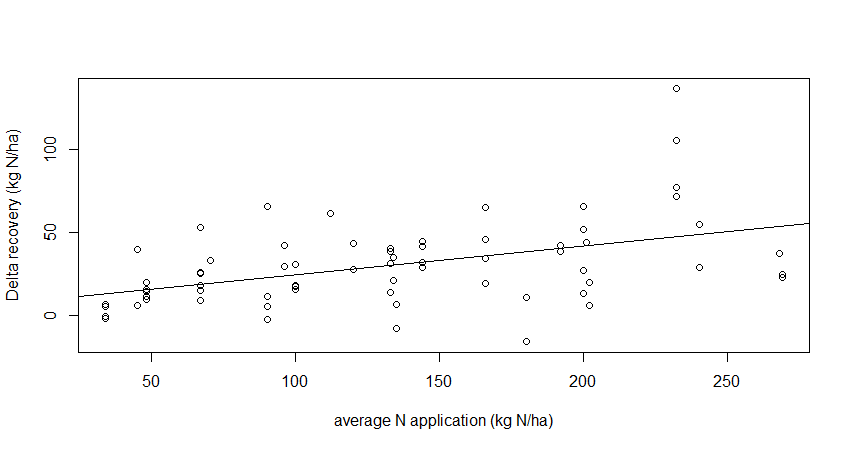

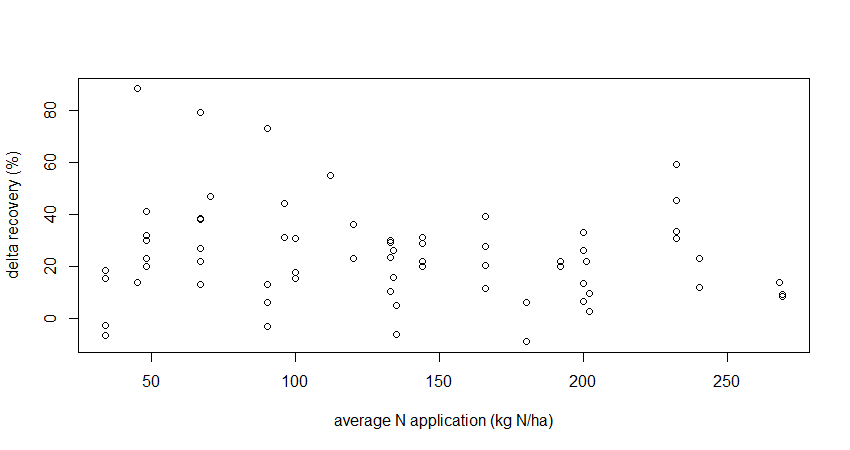

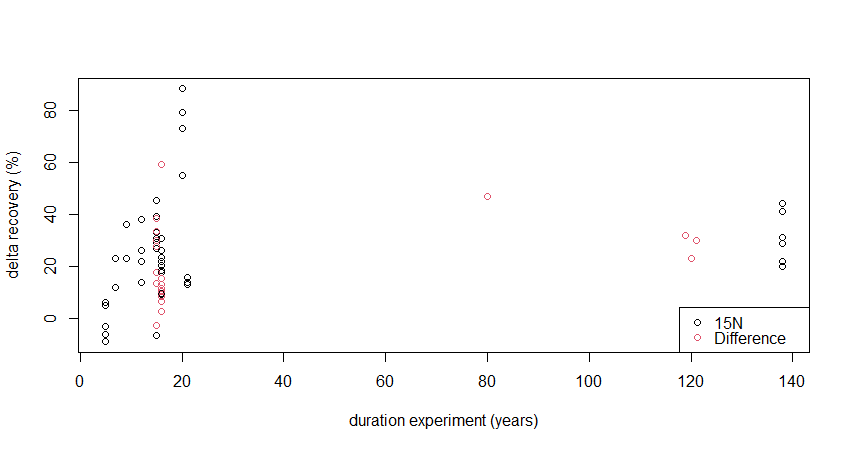


*SI-Figure 2: Influence of experiment duration and average N application on ∆RE (%). The dots indicate individual observations. Please note the change in units between the middle and the lower graph: the y-axis of the middle graph shows ∆RE in percentage of N applied while in the lower graph, ∆RE is expressed in kg N/ha. In the lower graph, there is a significant positive correlation between N application rate and ∆RE (P<0.01).*

| **Name experiment** | **Set-up** |
| --- | --- |
| Broadbalk | Long-term winter wheat cropping and fertilisation experiment since 1843. Main fertiliser treatments established in 1852 (Rothamsted Research, 2021). 15N microplots are from 1980-1983. |
| Ropsley | Between 1978 and 1990: crop rotation. After 1990: only winter wheat. Continuous fertilisation treatments with 0N and +N micro-plots established in 1992 and 1993. |
| Oklahoma-222 and 502 | Two continuous winter wheat cropping and fertilisation experiments since 1969 and 1970 respectively. 15N microplots were established in 1988. |
| Monmouth | Continuous maize cropping and fertilisation experiment since 1983. 15N microplots were established in 1994-1996. |
| Salisbury | Medium long-term experiment with continuous maize and fertilisation since 1973. Two treatments were tested: Ploughing and minimum tillage. Data from both tillage systems were included in this study. 15N microplots were established in 1974. |
| Iowa-central and southern | Continuous maize and fertilisation experiment since 1999. 0N and +N micro-plots were established in 2015. |
| Kiel | Long-term experiment since 1991, with a crop rotation including barley, winter wheat, and oilseed rape. Mineral fertiliser was applied in several splits. Only data from barley and winter wheat was used. 15N microplots were first established in 1996. |
| Hoosfield | Long-term spring barley fertilisation experiment since 1852. Since 1968 every original main plot fertiliser treatment was split into four subplots with different N rates, rotating annually since 1981 (Rothamsted Research, 2015). |
| Bad Lauchstädt | Long-term experiment since 1902, with changing crop rotation and fertiliser treatment. The average fertiliser rate was 70.38 kg N ha^-1^ between 1902 and 1977. Since 1978 part of the experiment was split into 5 new fertiliser treatments. |

*SI-Table 1: Setup of included experiments*

Rothamsted Research (2021) Broadbalk Wheat Experiment plan and cropping 1968-2017. Electronic Rothamsted Archive, Rothamsted Research. 10.23637/rbk1-plan1968-2017-01

Rothamsted Research (2015) Hoosfield spring barley experiment plans and fertilizer treatments, 1968-2000. Electronic Rothamsted Archive, Rothamsted Research. 10.23637/rhb2- plans1968-2000-01

Data obtained from LTEs Hoosfield and Bad Lauchstad did not allow for the “regular” subplot method as described in the main manuscript. Instead of a newly imposed subplot, there were complete alterations in the design of the experiment which allowed for a similar, but slightly different calculation of *∆RE*.

${\Delta RE}_{Hoosfield}=\left( \frac{U_{48,48}-U_{0, 0}}{48} \right)-\left( \frac{U_{48,48}-U_{0,48}}{48} \right)$ (SI-Equation 1)

*With:*

*U_48,48 :_ N uptake in plot which originally received 48 kg N ha^-1^, and now (in sampling year) receives 48 kg N ha^-1^.*

*U_0,0_: N uptake in plot which originally received 0 kg N ha^-1^, and now (in sampling year) receives 0 kg N ha^-1^.*

*U_0,48_: N uptake in plot which originally received 48 kg N ha^-1^, and now (in sampling year) receives 0 kg N ha^-1^.*

*48: Average N application rate before 1968 (kg N ha^-1^).*

${\Delta RE}_{Bad Lauchstädt}= \frac{U_{0,70}- U_{0,0}}{70.38}$ (SI-Equation 2)

*With:*

*U_0,70_: N uptake in plot which originally received 70.38 kg N ha^-1^, and now (in sampling year) receives 0 kg N ha^-1^*

*U_0,0_: N uptake in plot which originally received 0 kg N ha^-1^, and now (in sampling year) receives 0 kg N ha^-1^*

*70.38: Average N application rate between 1902 and 1977 (kg N ha^-1^).*


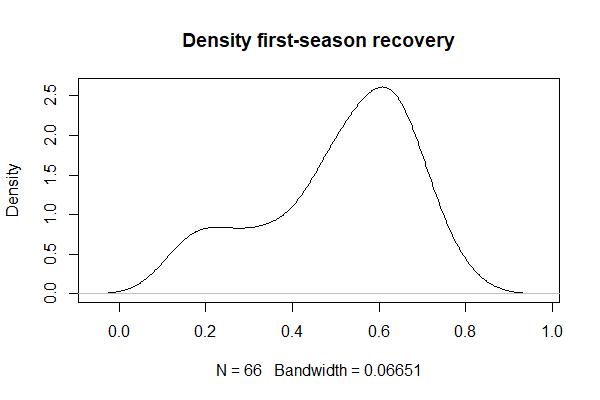

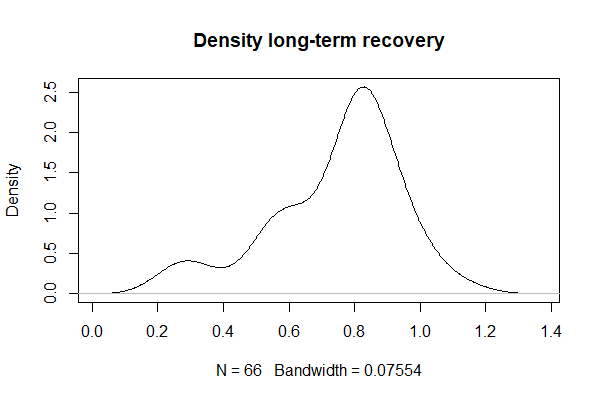

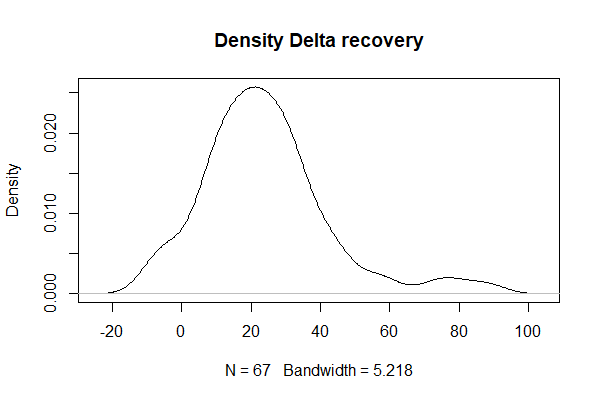


*SI-Figure 3: Density plots of RE^1ST^, RE^LT^ and ∆RE, showing the data distribution. The value for RE^1ST^, RE^LT^ and ∆RE is given on the x axis; the density on the y axis. For the density graph of ∆RE, sample size is 67 because Bad Lauchstädt is also included.*

**
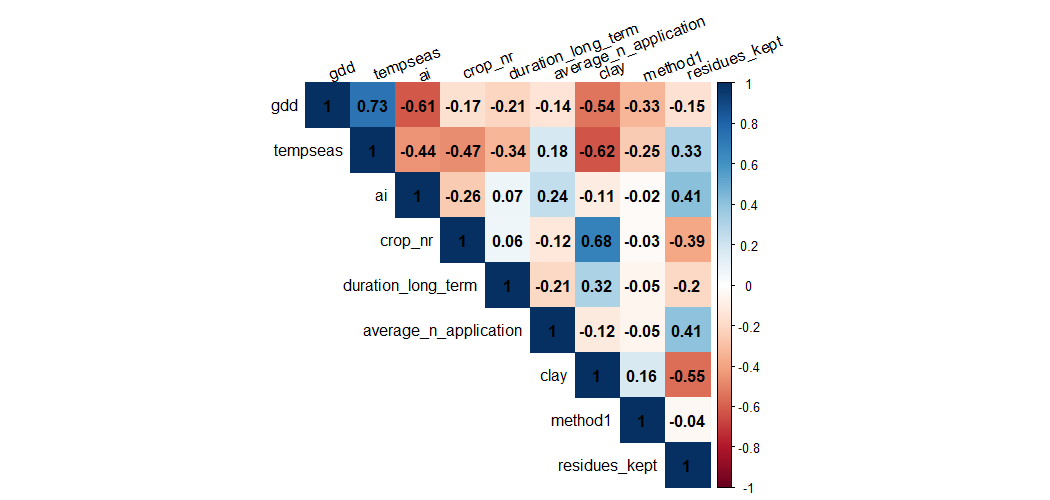
**

*SI-Figure 4: Correlation matrix of all co-variables included in the full model (equation 5). Numbers in black represent the correlation coefficient (r), which indicates severe collinearity when r>0.9. A white colour represents no correlation, a dark red or dark blue colour indicates severe collinearity. Gdd = growing degree days, tempseas = temperature seasonality, ai = aridity index, crop_nr = crop type, duration_long_term = experiment duration, average_n_application = average N application, clay = soil clay content, method1 = method, residues_kept = crop residue retention.*


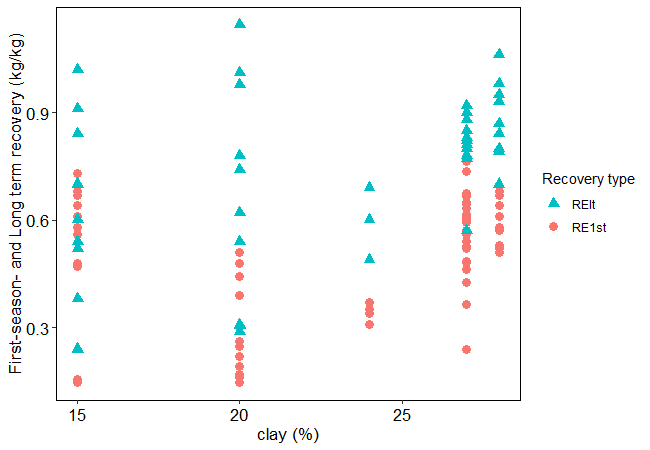


*SI-Figure 5: Influence of soil clay content on RE^LT^ and RE^1ST^ . The dots indicate individual observations, the lines indicates a mixed effect model of RE^LT and 1ST^ ~ clay content +* *ε. Please note that, despite the seemingly positive slopes, both slopes are not significantly different from 0. Bad Lauchstädt is excluded from the graph as it did not allow for separate calculation of RE^LT^ and RE^1ST^.*

**
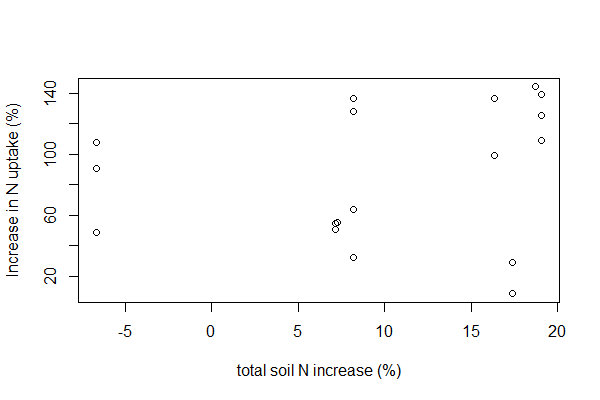
**

*SI-Figure 6: Difference in soil N uptake (%) between control and fertilised plots against related total soil N difference between control and fertilised plot (%).*

*SI-Table 2: Distribution of datapoints among crop type and experimental duration*

Rothamsted Research (2021) Broadbalk Wheat Experiment plan and cropping 1968-2017. Electronic Rothamsted Archive, Rothamsted Research. 10.23637/rbk1-plan1968-2017-01

Rothamsted Research (2015) Hoosfield spring barley experiment plans and fertilizer treatments, 1968-2000. Electronic Rothamsted Archive, Rothamsted Research. 10.23637/rhb2- plans1968-2000-01

| *Crop/Duration* | 5-10 years | 11-15 years | 16-25 years | >26 years |
| --- | --- | --- | --- | --- |
| Winter wheat | 2 | 14 | 21 | 10 |
| Maize | 6 | 4 | 4 |  |
| Barley | 2 |  |  | 3 |
| Rotation |  |  |  | 1 |

*SI-Table 3: Raw data from 9 long-term experiments in Europe and the USA. Exp=Experiment name; gdd=growing degree days; tempseas=temperature seasonality; aridity=aridity; duration = number of years; crop= crop type; N app=average N application; C N upt = N uptake in control plot; 15N upt = Uptake from short-term 15N experiment; T N upt= Total N uptake; ST RE= first season recovery; LT RE=long-term recovery; ΔRE=delta recovery; method = first-season recovery method(15N/subplot method); clay=soil clay percentage; resid=crop residue retention; soil pH=soil pH; carbon%=soil carbon percentage.*

| **exp** | **gdd** | **tempseas** | **aridity** | **duration** | **crop** | **N app** | **C N upt** | **15N upt** | **T N upt** | **ST RE** | **LT RE** | **ΔRE** | **method** | **cclay** | **resid** | **soil pH** | **carbon%** |
| --- | --- | --- | --- | --- | --- | --- | --- | --- | --- | --- | --- | --- | --- | --- | --- | --- | --- |
| Bad lauchstadt | 3242 | 6424 | 6719 | 80 | rot | 70 | 60 |  |  |  |  | 46.9 | subplot | 21 | no |  |  |
| Broadbalk | 3478 | 4999 | 9517 | 139 | ww | 96 | 31 | 60 | 132 | 0.61 | 1.06 | 44 | 15n | 28 | no | 7.75 | 1 |
| Broadbalk | 3478 | 4999 | 9517 | 139 | ww | 48 | 31 | 26 | 76 | 0.52 | 0.93 | 41 | 15n | 28 | no | 7.75 | 1 |
| Broadbalk | 3478 | 4999 | 9517 | 138 | ww | 96 | 30 | 60 | 121 | 0.64 | 0.95 | 31 | 15n | 28 | no | 7.75 | 1 |
| Broadbalk | 3478 | 4999 | 9517 | 139 | ww | 144 | 31 | 79 | 152 | 0.53 | 0.84 | 31 | 15n | 28 | no | 7.75 | 1 |
| Broadbalk | 3478 | 4999 | 9517 | 138 | ww | 144 | 30 | 96 | 171 | 0.68 | 0.98 | 29 | 15n | 28 | no | 7.75 | 1 |
| Broadbalk | 3478 | 4999 | 9517 | 139 | ww | 192 | 31 | 112 | 184 | 0.57 | 0.8 | 22 | 15n | 28 | no | 7.75 | 1 |
| Broadbalk | 3478 | 4999 | 9517 | 141 | ww | 144 | 29 | 87 | 143 | 0.58 | 0.79 | 22 | 15n | 28 | no | 7.75 | 1 |
| Broadbalk | 3478 | 4999 | 9517 | 138 | ww | 48 | 30 | 24 | 64 | 0.51 | 0.7 | 20 | 15n | 28 | no | 7.75 | 1 |
| Broadbalk | 3478 | 4999 | 9517 | 138 | ww | 192 | 30 | 121 | 197 | 0.67 | 0.87 | 20 | 15n | 28 | no | 7.75 | 1 |
| Broadbalk | 3478 | 4999 | 9517 | 140 | ww | 144 | 23 | 96 | 149 | 0.68 | 0.87 | 20 | 15n | 28 | no | 7.75 | 1 |
| Hoosfield | 3478 | 4999 | 9517 | 119 | b | 48 | 17 |  | 46.7 | 0.30 | 0.62 | 32 | subplot | 20 | no | 7.2 | 0.9 |
| Hoosfield | 3478 | 4999 | 9517 | 121 | b | 48 | 13 |  | 50.9 | 0.48 | 0.78 | 30 | subplot | 20 | no | 7.2 | 0.9 |
| Hoosfield | 3478 | 4999 | 9517 | 120 | b | 48 | 23 |  | 52.5 | 0.39 | 0.62 | 23 | subplot | 20 | no | 7.2 | 0.9 |
| Iowa-central | 3835 | 10863 | 8297 | 16 | m | 202 | 69 | 89 | 178 | 0.44 | 0.54 | 9.8 | 15n | 20 | yes | 6.0 | 1.9 |
| Iowa-central | 3835 | 10863 | 8297 | 16 | m | 202 | 69 | 89 | 178 | 0.51 | 0.54 | 2.9 | subplot | 20 | yes | 6.0 | 1.9 |
| Iowa-southern | 3956 | 10561 | 8782 | 16 | m | 269 | 45 | 40 | 108 | 0.15 | 0.24 | 9.1 | 15n | 15 | yes | 6.0 | 2.2 |
| Iowa-southern | 3956 | 10561 | 8782 | 16 | m | 269 | 45 | 40 | 108 | 0.16 | 0.24 | 8.4 | subplot | 15 | yes | 6.0 | 2.2 |
| Kiel | 3071 | 5848 | 12654 | 9 | ww | 120 | 72 | 80 | 194.9 | 0.67 | 1.02 | 36 | 15n | 15 | yes | 6.5 | 1.3 |
| Kiel | 3071 | 5848 | 12654 | 7 | b | 120 | 48 | 73 | 148.9 | 0.61 | 0.84 | 23 | 15n | 15 | yes | 6.5 | 1.3 |
| Kiel | 3071 | 5848 | 12654 | 9 | ww | 240 | 72 | 164 | 290.6 | 0.68 | 0.91 | 23 | 15n | 15 | yes | 6.5 | 1.3 |
| Kiel | 3071 | 5848 | 12654 | 7 | b | 240 | 48 | 116 | 192 | 0.48 | 0.6 | 12 | 15n | 15 | yes | 6.5 | 1.3 |
| Monmouth | 4103 | 10188 | 8713 | 12 | m | 67 | 75 | 21 | 121 | 0.31 | 0.69 | 38 | 15n | 24 | no |  |  |
| Monmouth | 4103 | 10188 | 8713 | 12 | m | 134 | 75 | 45 | 156 | 0.34 | 0.6 | 26 | 15n | 24 | no |  |  |
| Monmouth | 4103 | 10188 | 8713 | 12 | m | 201 | 75 | 75 | 195 | 0.37 | 0.6 | 22 | 15n | 24 | no |  |  |
| Monmouth | 4103 | 10188 | 8713 | 12 | m | 268 | 75 | 95 | 206 | 0.35 | 0.49 | 14 | 15n | 24 | no |  |  |
| Oklahoma-222 | 5745 | 8970 | 6627 | 21 | ww | 134 | 21 | 15 | 52 | 0.11 | 0.23 | 12 | 15n | 20 | no | 5.8 | 0.6 |
| Oklahoma-222 | 5745 | 8970 | 6627 | 21 | ww | 45 | 21 | 5 | 30 | 0.11 | 0.2 | 9 | 15n | 20 | no | 5.8 | 0.6 |
| Oklahoma-222 | 5745 | 8970 | 6627 | 21 | ww | 90 | 21 | 10 | 39 | 0.11 | 0.2 | 9 | 15n | 20 | no | 5.8 | 0.6 |
| Oklahoma-502 | 5373 | 9408 | 5672 | 20 | ww | 90 | 26 | 15 | 85 | 0.16 | 0.65 | 49 | 15n | 20 | no | 5.7 | 0.6 |
| Oklahoma-502 | 5373 | 9408 | 5672 | 20 | ww | 112 | 26 | 14 | 80 | 0.13 | 0.48 | 35 | 15n | 20 | no | 5.7 | 0.6 |
| Oklahoma-502 | 5373 | 9408 | 5672 | 20 | ww | 45 | 26 | 8 | 61 | 0.18 | 0.77 | 59 | 15n | 20 | no | 5.7 | 0.6 |
| Oklahoma-502 | 5373 | 9408 | 5672 | 20 | ww | 67 | 26 | 10 | 72 | 0.15 | 0.69 | 54 | 15n | 20 | no | 5.7 | 0.6 |
| Ropsley | 3340 | 4983 | 9111 | 15 | ww | 232 | 41 | 121 | 231 | 0.36 | 0.82 | 45.5 | 15n | 27 | no | 6.8 | 1.3 |
| Ropsley | 3340 | 4983 | 9111 | 15 | ww | 200 | 41 | 108 | 203 | 0.48 | 0.81 | 32.9 | 15n | 27 | no | 6.8 | 1.3 |
| Ropsley | 3340 | 4983 | 9111 | 15 | ww | 166 | 41 | 101 | 193 | 0.53 | 0.92 | 39.3 | 15n | 27 | no | 6.8 | 1.3 |
| Ropsley | 3340 | 4983 | 9111 | 16 | ww | 34 | 37 | 16 | 64 | 0.62 | 0.8 | 18.5 | 15n | 27 | no | 6.8 | 1.3 |
| Ropsley | 3340 | 4983 | 9111 | 15 | ww | 133 | 41 | 82 | 161 | 0.61 | 0.9 | 29.2 | 15n | 27 | no | 6.8 | 1.3 |
| Ropsley | 3340 | 4983 | 9111 | 15 | ww | 67 | 41 | 32 | 95 | 0.54 | 0.81 | 27.0 | 15n | 27 | no | 6.8 | 1.3 |
| Ropsley | 3340 | 4983 | 9111 | 16 | ww | 232 | 37 | 147 | 231 | 0.52 | 0.83 | 30.8 | 15n | 27 | no | 6.8 | 1.3 |
| Ropsley | 3340 | 4983 | 9111 | 15 | ww | 100 | 41 | 53 | 118 | 0.46 | 0.77 | 30.8 | 15n | 27 | no | 6.8 | 1.3 |
| Ropsley | 3340 | 4983 | 9111 | 16 | ww | 67 | 37 | 37 | 89 | 0.56 | 0.78 | 21.9 | 15n | 27 | no | 6.8 | 1.3 |
| Ropsley | 3340 | 4983 | 9111 | 16 | ww | 200 | 37 | 129 | 203 | 0.57 | 0.83 | 26.0 | 15n | 27 | no | 6.8 | 1.3 |
| Ropsley | 3340 | 4983 | 9111 | 16 | ww | 133 | 37 | 86 | 154 | 0.65 | 0.88 | 23.5 | 15n | 27 | no | 6.8 | 1.3 |
| Ropsley | 3340 | 4983 | 9111 | 16 | ww | 166 | 37 | 107 | 178 | 0.64 | 0.85 | 20.5 | 15n | 27 | no | 6.8 | 1.3 |
| Ropsley | 3340 | 4983 | 9111 | 16 | ww | 100 | 37 | 57 | 119 | 0.65 | 0.82 | 17.5 | 15n | 27 | no | 6.8 | 1.3 |
| Ropsley | 3340 | 4983 | 9111 | 15 | ww | 34 | 41 | 12 | 61 | 0.63 | 0.57 | -6.4 | 15n | 27 | no | 6.8 | 1.3 |
| Ropsley | 3340 | 4983 | 9111 | 15 | ww | 232 | 41 | 121 | 231 | 0.49 | 0.82 | 33.4 | subplot | 27 | no | 6.8 | 1.3 |
| Ropsley | 3340 | 4983 | 9111 | 15 | ww | 200 | 41 | 108 | 203 | 0.68 | 0.81 | 13.5 | subplot | 27 | no | 6.8 | 1.3 |
| Ropsley | 3340 | 4983 | 9111 | 15 | ww | 166 | 41 | 101 | 193 | 0.64 | 0.92 | 27.6 | subplot | 27 | no | 6.8 | 1.3 |
| Ropsley | 3340 | 4983 | 9111 | 16 | ww | 34 | 37 | 16 | 64 | 0.65 | 0.8 | 15.3 | subplot | 27 | no | 6.8 | 1.3 |
| Ropsley | 3340 | 4983 | 9111 | 15 | ww | 133 | 41 | 82 | 161 | 0.60 | 0.9 | 30.1 | subplot | 27 | no | 6.8 | 1.3 |
| Ropsley | 3340 | 4983 | 9111 | 15 | ww | 67 | 41 | 32 | 95 | 0.43 | 0.81 | 38.4 | subplot | 27 | no | 6.8 | 1.3 |
| Ropsley | 3340 | 4983 | 9111 | 16 | ww | 232 | 37 | 147 | 231 | 0.24 | 0.83 | 59.1 | subplot | 27 | no | 6.8 | 1.3 |
| Ropsley | 3340 | 4983 | 9111 | 15 | ww | 100 | 41 | 53 | 118 | 0.59 | 0.77 | 17.7 | subplot | 27 | no | 6.8 | 1.3 |
| Ropsley | 3340 | 4983 | 9111 | 16 | ww | 67 | 37 | 37 | 89 | 0.65 | 0.78 | 13.1 | subplot | 27 | no | 6.8 | 1.3 |
| Ropsley | 3340 | 4983 | 9111 | 16 | ww | 200 | 37 | 129 | 203 | 0.76 | 0.83 | 6.7 | subplot | 27 | no | 6.8 | 1.3 |
| Ropsley | 3340 | 4983 | 9111 | 16 | ww | 133 | 37 | 86 | 154 | 0.78 | 0.88 | 10.4 | subplot | 27 | no | 6.8 | 1.3 |
| Ropsley | 3340 | 4983 | 9111 | 16 | ww | 166 | 37 | 107 | 178 | 0.74 | 0.85 | 11.4 | subplot | 27 | no | 6.8 | 1.3 |
| Ropsley | 3340 | 4983 | 9111 | 16 | ww | 100 | 37 | 57 | 119 | 0.67 | 0.82 | 15.3 | subplot | 27 | no | 6.8 | 1.3 |
| Ropsley | 3340 | 4983 | 9111 | 15 | ww | 34 | 41 | 12 | 61 | 0.60 | 0.57 | -2.8 | subplot | 27 | no | 6.8 | 1.3 |
| Salisbury | 4928 | 8130 | 9732 | 5 | m | 90 | 60 | 58 | 123 | 0.64 | 0.7 | 6 | 15n | 15 | no | 6.2 |  |
| Salisbury | 4928 | 8130 | 9732 | 5 | m | 180 | 60 | 86 | 157 | 0.48 | 0.54 | 6 | 15n | 15 | no | 6.2 |  |
| Salisbury | 4928 | 8130 | 9732 | 5 | m | 135 | 60 | 75 | 141 | 0.56 | 0.6 | 5 | 15n | 15 | no | 6.2 |  |
| Salisbury | 4928 | 8130 | 9732 | 5 | m | 90 | 80 | 66 | 144 | 0.73 | 0.7 | -3 | 15n | 15 | no | 6.2 |  |
| Salisbury | 4928 | 8130 | 9732 | 5 | m | 135 | 80 | 78 | 150 | 0.58 | 0.52 | -6 | 15n | 15 | no | 6.2 |  |
| Salisbury | 4928 | 8130 | 9732 | 5 | m | 180 | 80 | 85 | 149 | 0.47 | 0.38 | -9 | 15n | 15 | no | 6.2 |  |
